# School for the Blind in Louisville

**Published:** 1842-04

**Authors:** 


### SCHOOL FOR THE BLIND IN LOUISVILLE.—PETTY MISREPRESENTATIONS.

In the *Boston Medical and Surgical Journal* for March the 2d, is the following paragraph from an anonymous Louisville letter, dated February 8th:

"We are just having a visit from Dr. Samuel G. Howe, the eminent superintendent of your Institution for the Blind. He is on a mission of benevolence, to establish a similar institution here. With four blind pupils he visited our Legislature at Frankfort, and there exhibited these redeemed children before the powers that be. The exhibition was satisfactory; the argument was convincing; and the General Assembly, with hardly a dissenting voice, voted to establish a school for the blind in Louisville, and granted ten thousand dollars to endow it, provided that this city would first raise the means to put it into operation. This would cost only about a thousand dollars, upon the plan proposed. We wanted no more of the State government. We can do the rest ourselves, and measures are now in train to obtain the preliminary funds here. Dr. Howe has exhibited these pupils here for several successive evenings, and demonstrated here, as well as at Frankfort, the practicability of educating the blind for usefulness and enjoyment. This matter is entirely new with us, and has excited great interest and drawn multitudes to the exhibitions; and without doubt, owing entirely to the benevolent energy of Dr. H., we shall soon have a small school in successful operation. It will be but a nucleus at first, to gather more funds and pupils. But we trust it will grow, so as to gather all the blind of the State within its folds."

Misstatements concerning the origin of public institutions should not be allowed to pass without correction. Under this sentiment we have extracted the foregoing, which displays, in a small way, more of systematic injustice than any corresponding number of lines which have lately fallen under our notice. In the beginning the writer endeavors to make the impression, that the "four blind pupils" were from Dr. Howe's school, when but *one* came with him from Boston, and the other three belong to the *Ohio Institution for the Blind*, where they have been exclusively taught (two of them being natives of that State), and whence they were sent by the Trustees of that school, in the care of its respected and enterprizing superintendent, Mr. Chapin, whose name is not mentioned. That gentleman, however, conducted two of the most interesting exhibitions made in Frankfort and Louisville, Dr. Howe not being present, at least in the latter. Again, the writer says "this matter is entirely new to us;" but what are the facts? They are these. In the winter preceding, 1840–41, a professor of the Medical Institute delivered once a week, in the Hall, a course of popular lectures on Physiology, and when he came to the sense of vision, devoted an evening to the method of instructing the blind. With the aid of Mr. Patton, a former pupil of the New England school, but at the time a resident of Louisville, the lecturer made an exhibition of the mode of teaching the blind, in reading, writing, arithmetic and geography, and concluded with an urgent appeal to the audience, one of the most numerous and intelligent ever assembled in the city, in favor of the establishment, in this city, of an institution for their instruction in letters and the mechanic arts. When the lecture closed, W. F. Bullock Esq., a member of the Legislature, came forward and declared that he would agitate the subject in the General Assembly as soon as the session should open; and before his departure he called on the lecturer and got a variety of reports and documents, by the aid of which he framed a bill, and sustained it by an able and eloquent speech. Without difficulty it passed the house of which he was a member, but was laid over in the other. At the opening of the next, the last session, this bill was called up. Meanwhile a friend of Dr. Howe in this city had written to him, and requested him to visit Kentucky, which he benevolently did, and on his way out, asked from the Ohio school the aid which was so promptly granted. So much for the assertion "that this matter is entirely new with us."

Dr. Howe's benevolent labors for the blind have not been so few and feeble, that his friends need claim for him what belongs to others; nor have they been so extraordinary as to justify an exclusive mention of his name, to the careful suppression of the names of those with whom he may happen to be associated.

